# Publisher’s Note

**DOI:** 10.1080/16549716.2019.1686864

**Published:** 2019-11-07

The following articles were originally published in issue 12(1). These have now been moved to issue 12(S1) (Special Issue: Antimicrobial Resistance):

FOREWORD

Making AMR history: a call to action

Tedros Adhanom Ghebreyesus

DOI: 10.1080/16549716.2019.1638144

ORIGINAL ARTICLE

Knowing antimicrobial resistance in practice: a multi-country qualitative study with human and animal healthcare professionals

Maddy Pearson and Clare Chandler

DOI: 10.1080/16549716.2019.1599560

METHODS FORUM

The ‘Drug Bag’ method: lessons from anthropological studies of antibiotic use in Africa and South-East Asia

Justin Dixon, Eleanor MacPherson, Salome Manyau, Susan Nayiga, Yuzana Khine Zaw,

Miriam Kayendeke, Christine Nabirye, Laurie Denyer Willis, Coll de Lima Hutchison and Clare I. R. Chandler

DOI: 10.1080/16549716.2019.1639388

Taylor & Francis apologises for this oversight.

